# Temporal and Spatial Diversity of Bacterial Communities in Coastal Waters of the South China Sea

**DOI:** 10.1371/journal.pone.0066968

**Published:** 2013-06-13

**Authors:** Jikun Du, Kai Xiao, Li Li, Xian Ding, Helu Liu, Yongjun Lu, Shining Zhou

**Affiliations:** 1 State Key Laboratory of Biocontrol, School of Life Sciences, Sun Yat-sen University, Guangzhou, China; 2 Department of Pharmacology, Guangdong Medical College, Dongguan, China; 3 Department of Clinical Laboratory, Shenzhen Shajing Affiliated Hospital of Guangzhou Medical University, Shenzhen, China; 4 South China Sea Fisheries Research Institute, Chinese Academy of Fishery Sciences, Guangzhou, China; Argonne National Laboratory, United States

## Abstract

Bacteria are recognized as important drivers of biogeochemical processes in all aquatic ecosystems. Temporal and geographical patterns in ocean bacterial communities have been observed in many studies, but the temporal and spatial patterns in the bacterial communities from the South China Sea remained unexplored. To determine the spatiotemporal patterns, we generated 16S rRNA datasets for 15 samples collected from the five regularly distributed sites of the South China Sea in three seasons (spring, summer, winter). A total of 491 representative sequences were analyzed by MOTHUR, yielding 282 operational taxonomic units (OTUs) grouped at 97% stringency. Significant temporal variations of bacterial diversity were observed. Richness and diversity indices indicated that summer samples were the most diverse. The main bacterial group in spring and summer samples was *Alphaproteobacteria*, followed by *Cyanobacteria* and *Gammaproteobacteria*, whereas *Cyanobacteria* dominated the winter samples. Spatial patterns in the samples were observed that samples collected from the coastal (D151, D221) waters and offshore (D157, D1512, D224) waters clustered separately, the coastal samples harbored more diverse bacterial communities. However, the temporal pattern of the coastal site D151 was contrary to that of the coastal site D221. The LIBSHUFF statistics revealed noticeable differences among the spring, summer and winter libraries collected at five sites. The UPGMA tree showed there were temporal and spatial heterogeneity of bacterial community composition in coastal waters of the South China Sea. The water salinity (*P*=0.001) contributed significantly to the bacteria-environment relationship. Our results revealed that bacterial community structures were influenced by environmental factors and community-level changes in 16S-based diversity were better explained by spatial patterns than by temporal patterns.

## Introduction

Bacteria are recognized as important agents in nutrient cycles and considered to be releasing inorganic matter through the decomposition of organic matter, thereby recycling nutrients to the phytoplankton [1–7]. Studies of bacteria have disclosed that marine bacterial populations are complex, widespread and often consisting of unidentified or uncultivated members [8–11]. Advances in molecular techniques and ecological genomics have greatly improved our understanding of the processes mediated by bacteria in the marine environments, including marine sediments, oligotrophic open sea, coastal temperate [12]. Although large populations of bacteria are well documented in coastal waters, their diversity and spatiotemporal variations remain largely unexplored.

As one of the most variable marine habitats, coastal waters are generally characterized by a high biodiversity and high primary production because these waters contain significant bacterial and nutritional inputs from terrestrial sources [13]. Numerous environmental factors have been suggested to influence the bacterial diversity (e.g. salinity, temperature and nutrients) [14]. Because of environmental heterogeneity, seasonal currents, anthropogenic effects, the coastal waters could harbor high bacterial diversity in response to geochemical and eutrophication gradients [15]. Long-term studies in the coastal ocean showed the robust seasonal patterns in species richness [16]. Marine bacteria demonstrate seasonal patterns in diversity with, generally, higher diversity during the winter than the summer in pelagic ecosystems [17]. It is possible that the ability of bacteria respond to seasonal variations could allow bacteria to respond to changing environmental conditions. Recently, the spatiotemporal profiles from ten samples of microbes in the coastal sediment of the South China Sea (SCS) were examined, showing that the microbial community structure was correlated with spatiotemporal variation [18].

The SCS is one of the largest marginal seas in the tropical Pacific Ocean, covers an area approximately 3,500,000 km^2^. It has a remarkable amount of biological diversity, including over 30% of the world’s coral reefs and many valuable fisheries [19]. With the rapid development of the tourism and the sudden increase in the population in the past 10 years, the coastal regions of the SCS are facing many ecological problems. However, to date, studies that describe the structure and composition of the bacterial communities in the coastal waters of the SCS are still limited due to the highly variable physical and biogeochemical conditions.

In this study, we aimed at exploring the composition of bacterial communities in the coastal waters of the SCS during different seasons using 16S rRNA gene sequences. Our primary focus was to find temporal and spatial patterns of bacterial communities and to gain an overall understanding of the bacterial diversity in this marine system. The results considerably extend our knowledge of the variations of bacterial communities responding to spatiotemporal variations. To our knowledge, this is the first comparison of the bacterial communities among different seasons in the coastal waters of the SCS by means of 16S rDNA sequences analysis.

## Materials and Methods

### Ethics Statement

No specific permits were required for the described field studies. The South China Sea Institute of Oceanology and Chinese Academy of Sciences issued the permission for each location. The location is not privately owned. The field studies did not involve endangered or protected species.

### Sampling Sites and sample collection

**Figure 1:**
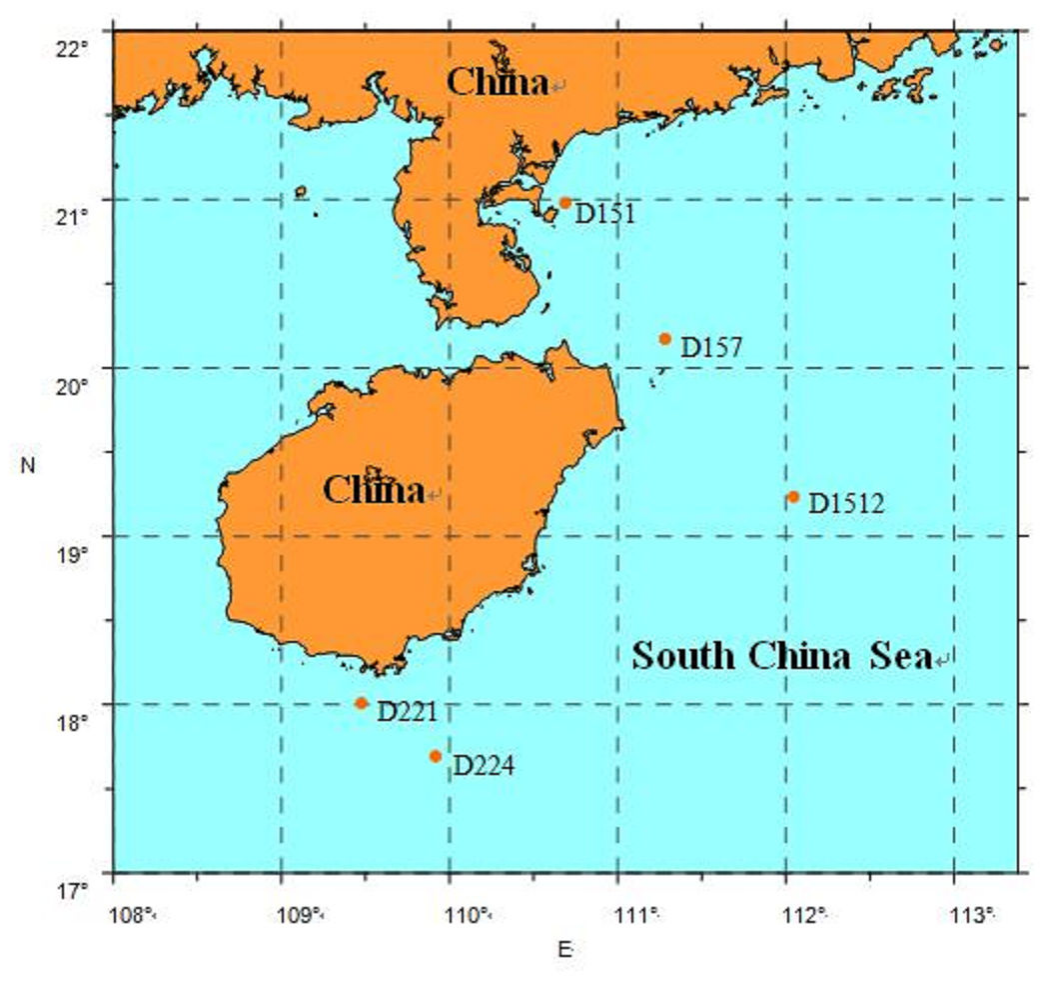
Map showing the sampling stations in the South China Sea.

Five sampling sites (D151, D157, D1512, D221, D224) were selected from the Hainan Island coastal area of the SCS (Figure 1). Water samples were collected from the five sites during summer (May) of 2006, spring (January) and winter (October) of 2007 by the Department of Guangzhou Marine Geological Survey. In total, we obtained 15 seawater samples. The characters used in the sample names indicate sampling seasons (SP, spring; S, summer; W, winter).

For each sample, 10 L of seawater were collected from surface (0-5 m) and pre-filtered through a 0.8-µm filter to separate free-living microorganisms. The filtrates were passed through a 0.2-µm filter. Then the 0.2-µm filter was immediately stored in sterile bags and frozen at -20°C before being processed. DNA samples were extracted under sterile conditions and to avoid cross contamination the manipulation of each sample was performed separately.

### Environmental parameters

Chemical data were determined in triplicates by standard oceanographic methods. Ambient water temperature and salinity was determined at the moment of sample collection using a hand-held refractometer (Leica). Other parameters were measured in the laboratory. Total organic carbon (TOC), total nitrogen (TN) was determined in accordance with the methods described by Grasshoff et al [20].

### DNA extraction and 16S rRNA gene amplification by PCR

Total genomic DNA was extracted from the filters using the bacteria genomic extraction kit (TianGen, Beijing) according to the manufacturer’s instructions. Genomic DNA of each sample was extracted induplicate. The 16S rRNA genes were amplified from the DNA templates by PCR with the universal primers Uni515F (5’ –GTGYCAGCMGCCGCGCGGTAA-3’) and Uni1406R (5’-TGACGGGCGGTGTGTRCAA-3’) [18]. PCR was performed in 25 µL reaction mixtures (1 × PCR buffer, 0.5mM dNTP, 1.25 U LA Taq polymerase [Takara], 1 ng of each primer and 100 ng of DNA template). To screen for potential contamination of PCR reagents, a negative PCR control using H_2_O instead of a DNA template was used. PCR amplification began with a 5 min denaturing step at 94 °C; followed by 26 cycles of 94°C 45 s, 50°C 45 s, 72°C 2 min; The final cycle was an extension at 72°C for 10 min. PCR products with expected sizes were excised from agarose gel after electrophoresis. The products were purified using EZNA Cycle-Pure Kits (Omega). The purity of the PCR amplifications was assessed by 1% (w/v) agarose gel electrophoresis.

### Construction of 16S rRNA gene clone libraries and amplified ribosomal DNA restriction analysis (ARDRA)

PCR products were cloned into the PMD20-T vectors as described by the manufacturer (Invitrogen, CA), and then transformed into chemically competent *E. coli* DH5α. Positive colonies for the blue-white colony screen used for the vector were picked. Approximately 100 white clones for each library were randomly selected for the 16S rRNA-ARDRA assay. The inserted 16S rDNA sequences were amplified by PCR with M13+/- primers. The 25 µL PCR reaction mixture contained 2.5 µL 10 × PCR buffer, 0.5 µL dNTP (2.5 mM), 1.25 U LA Taq polymerase, 0.5 µL each primer (10 µM) and 2 µL recombinant plasmid from each white clone. PCR amplification began with a 5 min denaturing at 94°C, and followed by 30 cycles of 94°C 30 s, 55°C 30 s, and 72°C 90 s; The final cycle was an extension at 72°C for 10 min. The resulting PCR products were further used for ARDRA analysis. All clones with both the same *Hae*Ⅲ and the same *Taq*Ⅰrestriction patterns were assigned to one ARDRA pattern [21].

### Sequencing and Phylogenetic Analyses

Representative clone of each ARDRA pattern was sequenced (Sangon Biological Engineering Technology and Services, China). All sequences obtained were edited to exclude the vector and the primer sequences, and checked for chimerical structures using the CHECK-CHIMERA program on the Ribosomal Database Project [22]. All sequences that were free of chimeras were compared with those in the GenBank database (http://www.ncbi.nlm.nih.gov/BLAST/) and the Ribosomal Database Project II (http://rdp.cme.msu.edu) using the Basic Local Alignment Search Tool (BLAST) to identify known sequences with a high degree of similarity. Meanwhile, the sequences were clustered as OTUs at an overlap identity cutoff of 97% by MOTHUR software [23]. One representative sequence from each dominant OTU was manually complied and aligned with their closest neighbors from GenBank database using Clustal X [24]. Phylogenetic tree including the representative sequences and their closest neighbors was constructed by neighbor-joining algorithm based on Jukes-Cantor-corrected distances in MEGA 4.0 [25]. The branches of the resultant tree were evaluated by bootstrap analysis based on 1000 replicates.

### Statistical methods for community analysis

Based on the result of ARDRA analysis, the coverage of the constructed 16S rRNA gene libraries were calculated using *C* = [1-(n/N)] according to Good (1953) [26], where *n* is the number of sequence types that occur only once in the library and *N* is the total number of clones examined. All the sequenced clones from the libraries were used for further analysis. Rarefaction analysis was conducted using MOTHUR program, Chaol richness estimates and Shannon-Weaver diversity index were calculated to further assess bacterial diversity and richness. The LIBSHUFF analysis was performed for pair-wise comparisons in each library to determine the significance of differences between clone libraries using the LIBSHUFF function available in MOTHUR, and *P* value was estimated by 10, 000 random permutations of sequences between libraries.

Bacterial community similarity analyses were conducted by the unweighted pair group method with arithmetic (UPGMA) algorithm according to the Yue & Clayton theta structural diversity measure using the MOTHUR software at OTU definition of 0.03 [23], which measures the molecular evolutionary distances of the sequences and can compare the evolutionary relationships among microbial communities that exist in different environments.

Correlations between bacterial communities and environmental factors were analyzed using the redundancy analysis (RDA) with the R package *vegan* [27]. The dominant OTUs were used as species input, and the environmental variables entered into the RDA were normalized (z-score transformation) [28]. Automatic forward selection with significant tests of Monte Carlo permutations were used to build the optimal models of bacteria-environment relationship (999 permutations) [27].

### Nucleotide sequence accession numbers

The nucleotide sequences determined in this study have been deposited at GenBank under accession numbers: EU181973-EU182214.

## Results

### Environmental parameters of the study sites

Abiotic parameters for each sampling site are shown in Table 1. Temperature varied from 15.1 to 28.5. The low salinity found at D151 site and D221 site is explained by the input of freshwater from terrestrial sources (*P*<0.01). The water geochemical parameters varied greatly between samples. For further analysis, the D151 site and D221 site were considered as coastal environments, and the D157 site, D1512 site and D224 site as offshore environments. Analysis of geochemical content showed the highest TOC and TN concentration at the coastal site D151, D221, which indicated a high input of organic carbon and the associated particles from terrestrial environment.

**Table 1:** Description and geochemical characteristics of the sampling stations.

**Sample**	**Location(E, N)**	**Temp (°C)**	**Salinity (‰)**	**TOC^a^ (mg/L)**	**TN^b^ (mg/L)**
D151SP	110°43′07", 20°54′22″	17.2	21.2	1.39	0.46
D151S	110°41′32″, 20°58′30″	26.2	16.9	2.05	0.65
D151W	110°41′45″, 20°57′44″	19.7	19.7	1.46	0.51
D157SP	111°17′03", 20°09’56"	16.5	27.9	1.52	0.55
D157S	111°17′13″, 20°10′03"	23.7	25.6	1.87	0.67
D157W	111°17′06", 20°10′13″	16.5	28.3	1.41	0.46
D1512SP	112°00’36", 19°13′15″	19.7	29.8	1.62	0.38
D1512S	112°03’06", 19°13′48″	27.2	27.9	1.70	0.60
D1512W	112°00’35", 19°13′11″	15.1	29.7	1.42	0.52
D221SP	109°29′28″, 17°59′55″	20.6	23.4	1.29	0.41
D221S	109°28′51″, 18°00’15"	26.8	18.2	1.53	0.62
D221W	109°29′22″, 17°59′57″	25.6	20.8	1.47	0.53
D224SP	109°55′16″, 17°41′28″	19.8	24.7	1.49	0.51
D224S	109°55′18″, 17°41′21″	28.5	25.0	1.56	0.59
D224W	109°54′55″, 17°41′35″	24.9	22.6	1.39	0.60

^a^ total organic carbon;

^b^ total nitrogen

Environmental parameters varied greatly between samples collected in different seasons. The concentration of TOC in the station D151, for instance, was 1.39 in spring, 2.05 in summer and 1.46 in winter (*P*<0.01) (Table 1), indicating complicated biogeochemical processes and hydrodynamic conditions between seasons.

### Coverage and diversity of clone libraries

A total of 15 bacterial clone libraries were constructed for the five sample sites. Approximately 90-100 clones from each clone library were used for the ARDRA analysis. The coverage of the respective libraries ranged from 72% to 91% (Table 2), indicating the clone numbers screened in each library can exhibit the diversity of the sample site.

**Table 2:** Analyses of the 15 bacterial clone libraries in the South China Sea.

**Sample**	**No. of ARDRA patterns**	**No. of sequenced clones**	**No. of OTUs**	**Coverage (%)**	**H′^a^**	**Chao1**
D151SP	24	39	26	75.5%	3.26	351
D151S	21	42	23	78.4%	3.13	276
D151W	19	37	31	80.0%	3.43	496
D157SP	12	23	9	88.0%	2.20	45
D157S	18	34	26	80.8%	3.26	351
D157W	10	15	12	89.5%	2.48	78
D221SP	18	36	19	81.5%	2.94	190
D221S	15	37	19	84.0%	2.94	190
D221W	10	13	11	90.5%	2.40	66
D224SP	21	41	25	78.0%	3.22	325
D224S	17	41	24	82.4%	3.18	300
D224W	20	27	18	79.5%	2.89	171
D1512SP	12	23	2	88.0%	0.69	3
D1512S	27	63	38	72.0%	3.71	741
D1512W	13	20	15	87.0%	3.64	120

^a^ Shannon-Weaver diversity index (Hʹ = - ΣPi log Pi *N*).

The representative 491 clones from all the ARDRA patterns were sequenced. Rarefaction curves were drawn for the spring groups, summer groups and winter groups (Figure S1), which indicated that the major members of the community had been sampled, and there is likely a long tail of rare taxa which are not included in the study. Total number of representative clones analyzed for each sample varied between 13 and 63, with an average of 32. Based on a taxa cutoff set at 97% similarity, the sequences were further grouped into 282 OTUs. The number of OTUs for each sample ranged from 2 to 38 with average of 20. The Shannon diversity index and the Chao1 estimator of species diversity for each sample were calculated (Table 2). The lowest diversity indices in the spring libraries indicated that its microbial community was composed of a few phylotypes, while in the summer libraries, the diversity indices showed a higher level of species richness. Tables 2 also showed that, for the D151 site and D221 site, the species diversity was higher than that of other sites. The lowest species diversity was observed in the D1512 site.

### Bacterial community composition analysis of 16S rDNA clone libraries

**Figure 2:**
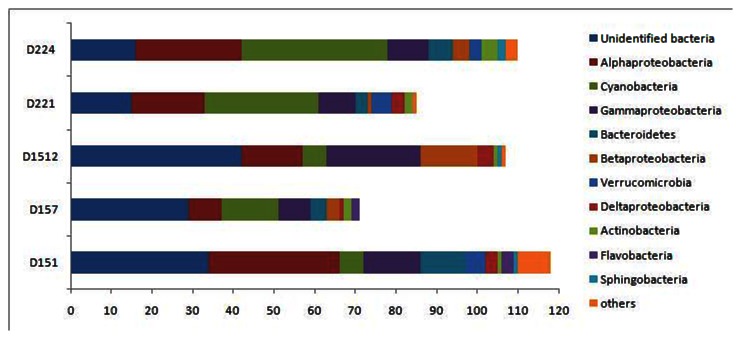
The spatial profiles of the bacterial community structure based on 16S rRNA gene clone libraries from the five sampling sites. D151, samples collected from D151 site; D157, samples collected from D157 site; D1512, samples collected from D1512 site; D221, samples collected from D221 site; D224, samples collected from D224 site.

**Figure 3:**
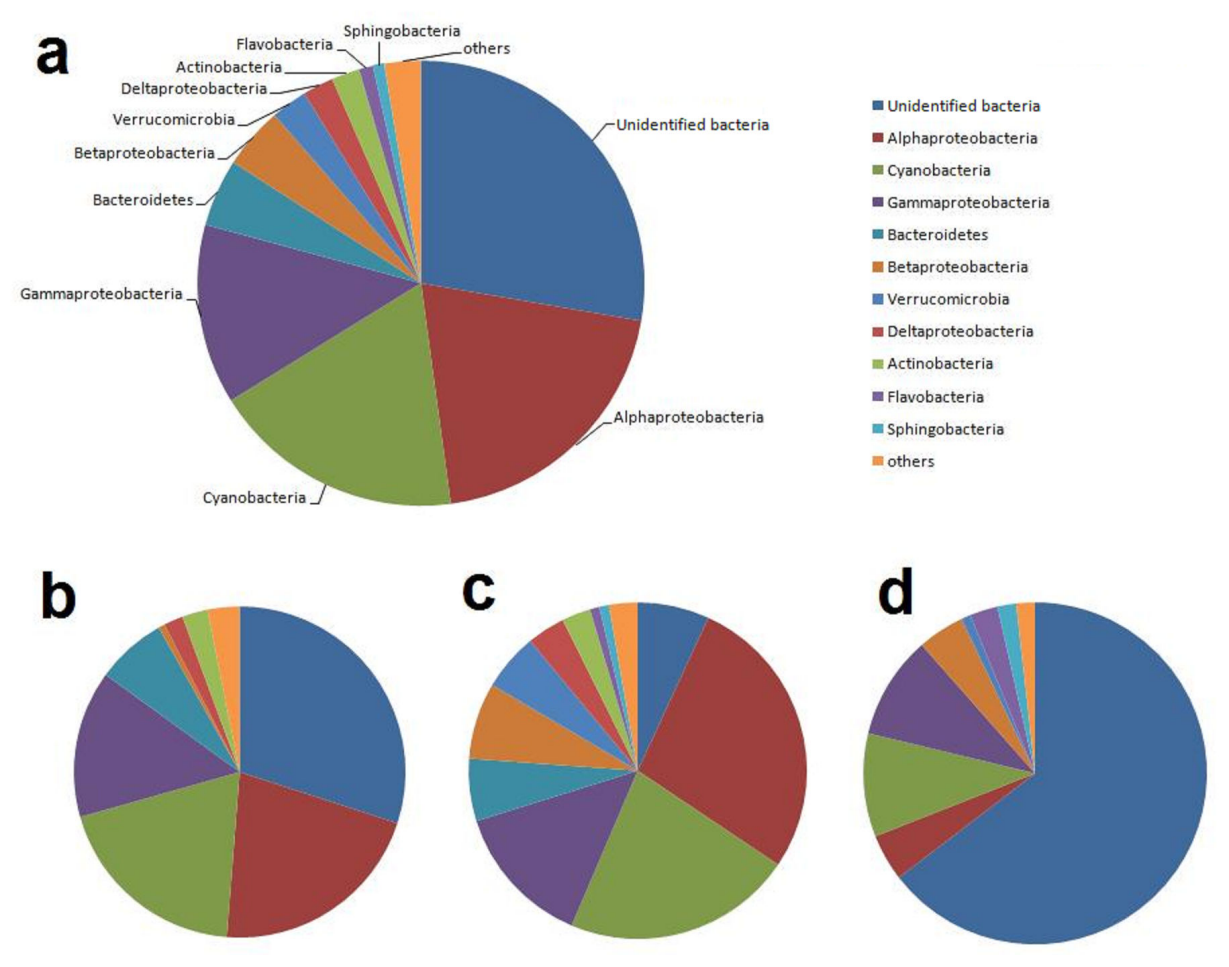
Pie charts of the relative abundance of the bacterial 16S rRNA gene clusters a, all the sequenced clones; b, water samples collected in spring; c, water samples collected in summer; d, water samples collected in winter.

The phylum composition of each clone library was shown in Figure 2 and Figure 3a. All of our sequenced clones fell into the nine major lineages of the bacterial domain: *Alpha-, Gamma-, Delta-, and Betaproteobacteria; Cyanobacteria; Bacteriodetes; Verrucomicrobia; Actinobacteria*; Unindentified bacteria. The percentage of 16S rDNA sequences from each group indicated that unindentified bacteria dominated D151, D157, D1512 sites, while *Cyanobacteria* dominated the D221, D224 sites. For the bacterial communities from each group, *Alphaproteobacteria* was the major group at D151, D221, D224 sites, while *Gammaproteobacteria* was the major group at D1512 site and *Cyanobacteria* was the major group at D157 site. *Actinobacteria* also occurred in all of the samples but was not abundant. *Bacteroidetes, Betaproteobacteria, Verrucomicrobia* and *Deltaproteobacteria* were detected in the clone libraries but not in all the sample sites. Others (*Flavobacteria, Sphingobacteria, Planctomycetes* and *Firmicutes*) were also detected as minor groups in a few of the samples

### Phylogenetic analysis of bacterial 16S rRNA genes across all sites and samples

**Figure 4:**
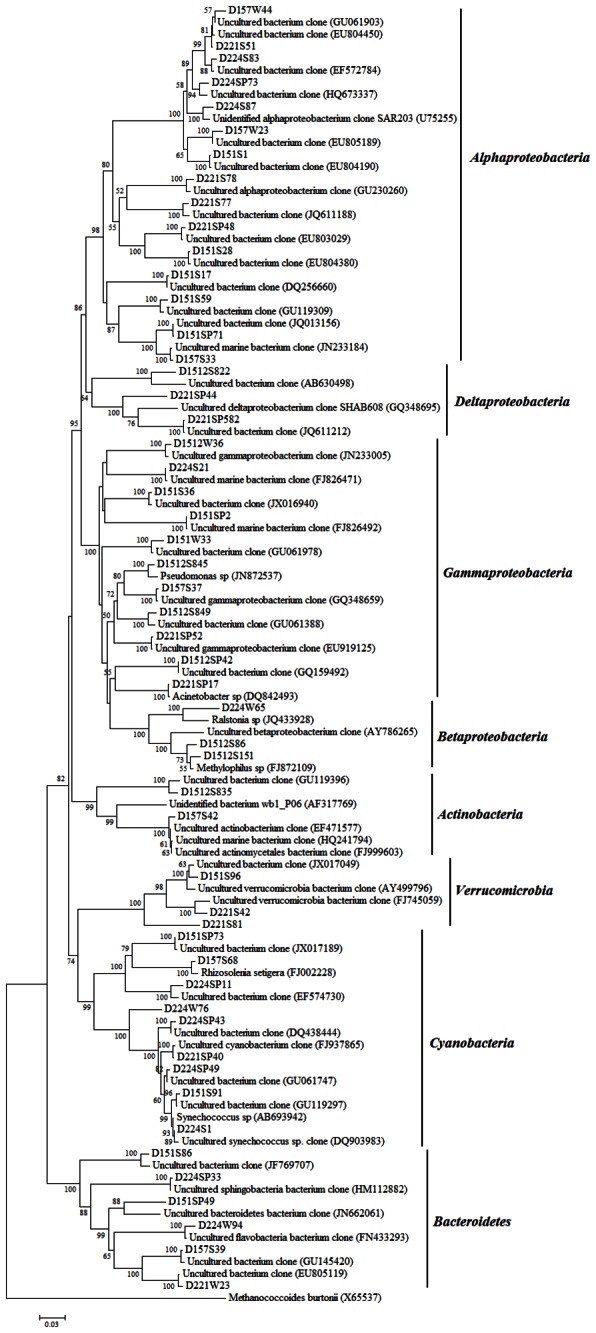
Phylogenetic tree based on analysis of the representative 16S rRNA gene sequences obtained from the 15 bacterial clone libraries. The tree was constructed using the neighbor-joining method in MEGA. Bootstrap analysis was conducted using 1000 replicates. Bootstrap values are shown for branches with > 50% bootstrap support.

A phylogenetic tree was constructed to show relationships between the dominant OTUs (52 OTUs, representing 334 sequences) and their closest neighbors (Figure 4). Based on the valid reference tree, eight different phyla were identified, with the majority of OTUs being classified as *Proteobacteria*, followed by *Cyanobacteria* , *Bacteroidetes*, *Verrucomicrobia* and *Actinobacteria*, respectively. The phylogenetic tree of dominant OTUs did not include any of these unidentified bacteria in Figure 3, which indicated the unidentified bacteria were rare taxa, and less dominant in the community.

A total of 32 OTUs representing 200 sequences were classified within the phylum *Proteobacteria* (Table 3). Four bacterial subphyla were identified: *Alphaproteobacteria*, *Betaproteobacteria*, *Gammaproteobacteria* and *Deltaproteobacteria*. The two predominant subphyla were *Alphaproteobacteria* and *Gammaproteobacteria*. Within the subphylum *Alphaproteobacteria* (15 OTUs, representing 102 sequences), OTUs were closely related to uncultured. The subphylum *Gammaproteobacteria* was comprised of 11 OTUs (76 sequences), most of them were related to the uncultured *Gammaproteobacteria*. The other two OTUs (22 sequences) were classified within the order *Acinetobacteria* (6 sequences), *Pseudomonas* (3 sequences), respectively. Six OTUs (22 sequences) were classified within the subphylum *Betaproteobacteria* and *Deltaproteobacteria*, respectively.

**Table 3:** Phylogenetic affiliation of library clones obtained from coastal waters of the South China Sea as deduced from BLAST searched.

**Representative Clones**	**No. of similar sequences**	**Phylogenetic ascription**	**Closest relative (accession number)**	**identity**
D157W44	3	Alphaproteobacteria	Uncultured bacterium clone S-5m-75(GU061903)	99%
D221S51	11	Alphaproteobacteria	Uncultured bacterium clone 6C232378(EU804450)	99%
D224S83	38	Alphaproteobacteria	Uncultured bacterium clone S23_883(EF572784)	99%
D224SP73	2	Alphaproteobacteria	Uncultured bacterium clone F9P41300_A04(HQ673337)	98%
D224S87	3	Alphaproteobacteria	Unidentified alpha proteobacterium clone SAR203(U75255)	98%
D157W23	2	Alphaproteobacteria	Uncultured bacterium clone(EU805189)	99%
D151S1	12	Alphaproteobacteria	Uncultured bacterium clone 6C232086(EU804190)	99%
D221SP48	4	Alphaproteobacteria	Uncultured bacterium clone 4C230433(EU803029)	99%
D151S28	2	Alphaproteobacteria	Uncultured bacterium clone 6C232292(EU804380)	99%
D221S78	4	Alphaproteobacteria	Uncultured alpha proteobacterium clone ARTE1_103(GU230260)	99%
D221S77	2	Alphaproteobacteria	Uncultured bacterium clone KSTye-PF1-B-003(JQ611188)	99%
D151S17	3	Alphaproteobacteria	Uncultured bacterium clone Fitz2_28(DQ256660)	99%
D151S59	2	Alphaproteobacteria	Uncultured bacterium clone Reef_N07(GU119309)	98%
D151SP71	3	Alphaproteobacteria	Uncultured bacterium clone DMS16SrDNA22(JQ013156)	99%
D157S33	11	Alphaproteobacteria	Uncultured marine bacterium clone IMS3D32(JN233184)	99%
D1512S822	5	Deltaproteobacteria	Uncultured bacterium clone MPB1-116(AB630498)	94%
D221SP582	2	Deltaproteobacteria	Uncultured bacterium clone KSTye-VF1-B-020(JQ611212)	99%
D221SP44	3	Deltaproteobacteria	Uncultured delta proteobacterium clone SHAB608(GQ348695)	92%
D1512S151	3	Betaproteobacteria	Uncultured beta proteobacterium clone 161GNFL6(AY786265)	92%
D1512S86	7	Betaproteobacteria	*Methylophilus* sp. Mim(FJ872109)	99%
D224W65	2	Betaproteobacteria	*Ralstonia* sp. LT3(JQ433928)	99%
D224S21	2	Gammaproteobacteria	Uncultured marine bacterium clone A6-5-63(FJ826471)	99%
D221SP17	6	Gammaproteobacteria	*Acinetobacter* sp. EN96(DQ842493)	99%
D151S36	5	Gammaproteobacteria	Uncultured bacterium clone HglFeb5F7(JX016940)	99%
D1512W36	3	Gammaproteobacteria	Uncultured gamma proteobacterium clone OS3SD61(JN233005)	99%
D151SP2	5	Gammaproteobacteria	Uncultured marine bacterium clone A6-5-84(FJ826492)	99%
D1512SP42	40	Gammaproteobacteria	Uncultured bacterium clone 16slp92-01d01.q1k(GQ159492)	99%
D151W33	2	Gammaproteobacteria	Uncultured bacterium clone S-DCM-17(GU061978)	98%
D1512S845	3	Gammaproteobacteria	*Pseudomonas* sp. SAP34_1(JN872537)	99%
D157S37	2	Gammaproteobacteria	Uncultured gamma proteobacterium clone SHAB561(GQ348659)	99%
D1512S849	6	Gammaproteobacteria	Uncultured bacterium clone CE1-5m-107(GU061388)	99%
D221SP52	2	Gammaproteobacteria	Uncultured gamma proteobacterium clone SW45(EU919125)	99%
D151S96	2	Verrucomicrobia	Uncultured bacterium clone HglFeb5G9m(JX017049)	98%
D221S81	2	Verrucomicrobia	Uncultured Verrucomicrobia bacterium clone Dover396(AY499796)	97%
D221S42	8	Verrucomicrobia	Uncultured Verrucomicrobiae bacterium clone SHWN (FJ745059)	97%
D224W76	2	*Cyanobacteria*	Uncultured *Synechococcus sp.* clone PR12 (DQ903983)	98%
D151SP73	3	*Cyanobacteria*	Uncultured bacterium clone HglFeb6C1m(JX017189)	99%
D157S68	2	*Cyanobacteria*	Rhizosolenia *setigera* isolate C22(FJ002228)	99%
D224SP11	2	*Cyanobacteria*	Uncultured bacterium clone S25_1074(EF574730)	97%
D221SP40	7	*Cyanobacteria*	Uncultured cyanobacterium clone MWLSA52(FJ937865)	99%
D224SP43	15	*Cyanobacteria*	Uncultured bacterium clone ECS-P7-C9(DQ438444)	99%
D224SP49	4	*Cyanobacteria*	Uncultured bacterium clone CEP-5m-60(GU061747)	99%
D151S91	4	*Cyanobacteria*	Uncultured bacterium clone Reef_G16(GU119297)	99%
D224S1	52	*Cyanobacteria*	*Synechococcus sp.*(AB693942)	99%
D157S42	2	Actinobacteria	Uncultured marine bacterium clone Sp02sw-15(HQ241794)	99%
D1512S835	13	Actinobacteria	Uncultured bacterium clone Reef_M14(GU119396)	99%
D151S86	5	Bacteroidetes	Uncultured bacterium clone REP6-45(JF769707)	98%
D224SP33	2	Bacteroidetes	Uncultured Sphingobacteria bacterium clone SHOF496	99%
D151SP49	2	Bacteroidetes	Uncultured Bacteroidetes bacterium clone LF8CBb87(JN662061)	91%
D224W94	3	Bacteroidetes	Uncultured Flavobacteria bacterium(FN433293)	98%
D157S39	2	Bacteroidetes	Uncultured bacterium clone BS035(GU145420)	99%
D221W23	2	Bacteroidetes	Uncultured bacterium clone 6C233107(EU805119)	99%

Nine OTUs representing 91 sequences were classified within the phylum *Cyanobacteria* . Two subphyla were identified: *Synechococcus* (5 OTUs, 82 sequences) and *Rhizosolenia* (3 OTUs, 7 sequences). In comparison with *Rhizosolenia*, *Synechococcus* has a high relative abundance. The less prominent 9 OTUs (28 sequences) were classified within the phylum *Verrucomicrobiae* and *Bacteroidetes*. The remaining two OTUs (15 sequences) were classified within the phylum *Actinobacteria*.

### Community structures of bacteria with seasons and between locations

The distribution of bacteria clones with seasons was represented in Figure 3a. Unidentified bacteria dominated the spring libraries, followed by *Alphaproteobacteria,* *Cyanobacteria* *, Gammaproteobacteria, Bacteroidetes, Actinobacteria, Delataproteobacteria* and *Betaproteobacteria* (Figure 3b). The unidentified bacteria were also the largest phylum in the winter libraries, followed by *Cyanobacteria* *, Gammaproteobacteria, Alphaproteobacteria, Betaproteobacteria, Actinobacteria, Flavobacteria, Sphingobacteria* and *Verrucomicrobia* (Figure 3d). While in the summer libraries, *Alphaproteobacteria* was the largest phylum, followed by *Cyanobacteria* , *Gammaproteobacteria, Betaproteobacteria, Bacteroidetes, Verrucomicrobia, Delataproteobacteria, Actinobacteria, Flavobacteria* and *Sphingobacteria* (Figure 3c) *.*

The partitioning of bacterial diversity among the three groups based on seasons was analyzed using LIBSHUFF analysis (10,000 randomizations). The result revealed that there were significant differences (*P*<0.008) in phylogenetic composition between all the three groups (Table 4), indicated an interesting partitioning of bacterial diversity responding to season variations. To identify the spatial variations, libraries were divided into five groups based on the five sample sites. The LIBSHUFF analysis (10,000 randomizations) was performed to determine statistically differences between the five groups (Table 5). The results showed that most of the groups were significantly different (*P*<0.0025), with the exception of the community structures of D157 and D224, D221 and D224.

**Table 4:** LIBSHUFF analysis of the community structures of spring samples, summer samples and winter samples at an OTU definition level of 97%.

	**Spring**		**Summer**		**Winter**	
	dCXYScore	*P* value*	dCXYScore	*P* value*	dCXYScore	*P* value*
Spring			0.001	0.036	0.012	<0.001
Summer	0.023	<0.001			0.025	<0.001
Winter	0.001	0.009	0.001	0.027		

* The significance values should be below the critical threshold (0.05/6 = 0.008)

**Table 5:** LIBSHUFF analysis of the community structures of samples collected at D151, D157, D1512, D221, D224 at an OTU definition level of 97%.

	**D151**		**D157**		**D1512**		**D221**		**D224**	
	dCXYScore	*P* value*	dCXYScore	*P* value*	dCXYScore	*P* value*	dCXYScore	*P* value*	dCXYScore	*P* value*
D151			0.009	<0.001	0.023	<0.001	0.008	<0.001	0.014	<0.001
D157	0.007	<0.001			0.002	0.0039	0.002	0.0035	0.002	0.0030
D1512	0.065	<0.001	0.014	<0.001			0.043	<0.001	0.022	<0.001
D221	0.001	0.0032	0.006	<0.001	0.005	<0.001			0.002	0.0056
D224	0.004	<0.001	0.003	0.0028	0.005	<0.001	0.003	0.0043		

* The significance values should be below the critical threshold (0.05/20 = 0.0025)

**Figure 5:**
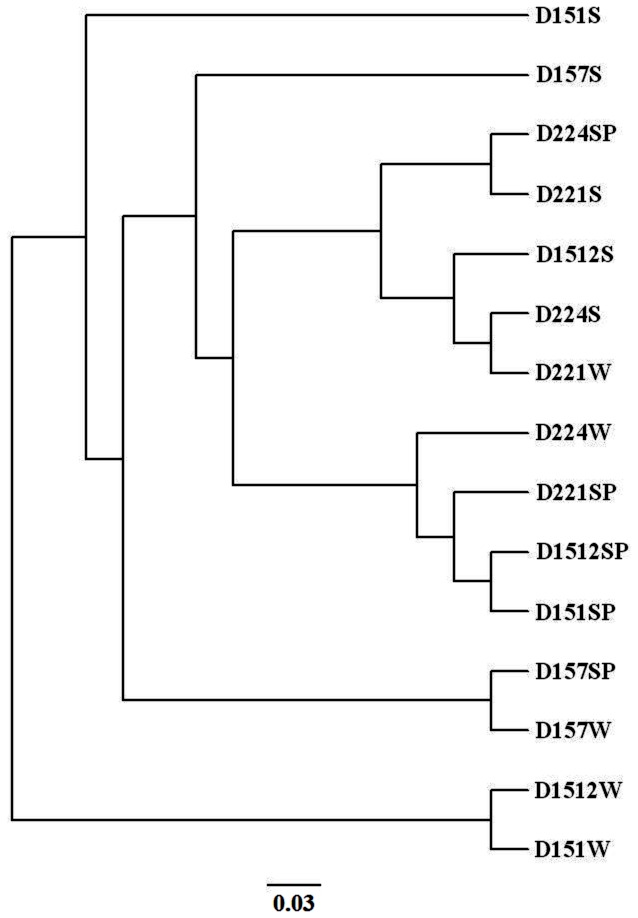
UPGMA cluster of the samples collected from the five locations in spring, summer and winter at OTU definition of 0.03.

The community assemblages were clustered using the UPGMA algorithm in the program MOTHUR at the OTU definition level of 97% (Figure 5). In the UPGMA tree, bacterial communities collected in spring and summer displayed less variation across the spatial profile, on the other hand, those collected in winter were more heterogeneous. The D221 and D224 samples collected at spring, summer and winter clustered with the D1512 sample collected at spring and summer (Figure 5). Unlike the spring and summer samples, the winter samples collected from D151, D157, D1512 were clearly separated from the spring and summer samples except for the D157SP sample (Figure 5). The D157SP and D157W samples were nearly identical, yet differed greatly from most of the other communities. The D151S sample was nearly as different from all of the other communities as D151W and D1512W samples.

### Relationships of environmental factors and bacterial community structures

**Figure 6:**
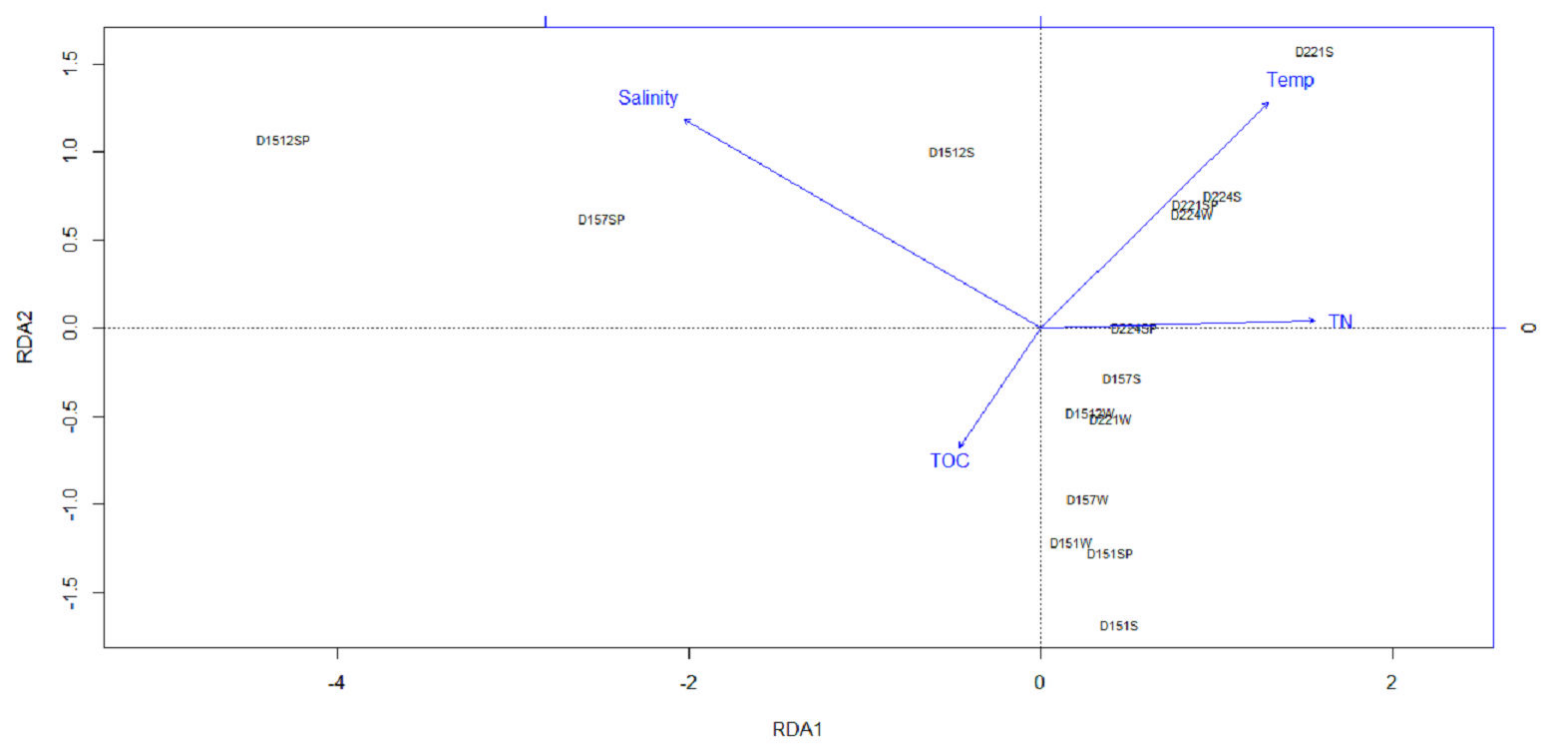
RDA ordination plots for the environmental parameters and the bacterial communities represented by 16S rRNA gene sequences.

RDA was used to determine how environmental parameters influenced the bacterial community structures, after initial analysis by detrended correspondence analysis (Figure 6). The first two RDA axes explained 64.0% of the cumulative variance of the bacteria-environment relationship. The water salinity (*P*=0.001) and TOC (*P*=0.089) contributed most to this distinction and contributed significantly to the bacteria-environment relationship. The concentration of salinity contributed to the distribution of the water samples, especially for D1512SP, D1512S, D157SP, while the separation of D221S, D221SP, D224S, D224W was a result of the temperature (Figure 6).

## Discussion

Our results demonstrate that the biodiversity of marine bacteria communities at the SCS exhibit distinctly spatial and temporal patterns. These patterns in distribution and abundance of marine bacteria were influenced by a variety of environmental variables. Our data and analyses show that marine bacterial diversity is higher in summer and that bacterial community composition exhibits a spatial gradient of increasing diversity from offshore to coastal sea (Table 2, Table 5). The UPGMA analysis for our samples showed that community-level changes in 16S-based diversity were better explained by spatial patterns than by temporal patterns (Figure 5, Table 4, Table 5). These patterns found in community composition can add new knowledge to bacterioplankton abundance and distribution in coastal marine ecosystems of the SCS.

In this study, although we have explored only a limited number of sets of OTUs at 97% sequence similarity, a number of strong patterns are apparent. The association with the environmental variables of temperature, salinity, nutrients and organic matter, suggest a large number of possible ecological mechanisms responsible for these patterns (Figure 6). Our sample location (D151, D157, D1512, D221, D224) off Hainan Island is within a system of currents that exhibit strong seasonality, with generally southwestward flow (China Coastal current) in spring, northeastward flow (western Pacific warm current) in summer, and weak southwestward flow in early winter [29]. Because the region northeast of our site is the Pearl River and the Jianjiang River estuaries, one might expect the China Coastal current from northeast to bring more eutrophic conditions and associated organisms of these estuaries in spring, whereas western Pacific warm current from southwest would be associated with more oligotrophic conditions and associated organisms. As higher rates of resource supply can potentially support larger numbers and more specialized kinds of organisms, the diversity of organism increases with increasing productivity [30,31]. It is likely that these seasonal hydrographic conditions and their influence on bacteria, contributed significantly to our observed patterns. Such variations in hydrography are reflected in several parameters we investigated, such as temperature, salinity and nutrients.

Our data showed bacterial diversity in the coastal waters (D151, D221) was higher than that in the offshore waters (D157, D1512, D224). As terrestrially impacted seawater has a higher concentration of particles than offshore seawater, which receives a low input of organic carbon and the associated particles, the environmental variables (e.g., nutrient, salinity) display lateral gradient patterns related with the distance to shore [32]. The geochemical variables suggested the primary production at the coastal regions (D151, D221) was higher than that at offshore regions (D157, D1512, D224) (Table 1, Table 2). The primary production of the coastal waters is known to commonly exceed the consumption of herbivores. Therefore, a large fraction of primary organic matter becomes available to consumers as detritus [33]. Most of this detritus is degraded by heterotrophic bacteria before entering higher trophic levels [34,35], and this results in the more diverse bacterial communities in the coastal waters than the offshore waters.

Both the coastal sites D151 and D221 exhibited similar spatial patterns with higher bacterial diversity than that of the offshore sites. However, the diversity of bacterial communities in the coastal site D151 and D221 showed different temporal patterns (Table 2), which might suggest the mixing of two seasonal currents was blocked by Hainan Island. As the blocking of the currents by Hainan Island, the temporal pattern of the coastal site D151 might be mainly contributed by China Coastal current, while that of the coastal site D221 might be mainly contributed by west Pacific warm current. In addition, the results of UPGMA analysis showed bacterial communities collected during spring and summer displayed less variation across the spatial profile (Figure 5), while those collected during summer were more heterogeneous (Figure 5, Table 4). Due to the block of currents by Hainan Island, samples collected in coastal site D151 clustered separately, while samples collected in coastal site D221 clustered together. Thus, our results indicate that spatial patterns show more heterogeneous than temporal patterns in the coastal waters of the SCS.

Past studies have found some similar results in comparison to those we report here. For example, Gilbert et al. [36] reported repeatable seasonal patterns occurred in surface water microbial community and the driver of this pattern was day length. Morris et al. [37] found certain bacterial groups tended to be more common during certain seasons. Fuhrman et al. [17] showed repeatable temporal pattern in distribution and abundance of microbial taxa was highly predictable. Their temporal pattern was most strongly correlated to parameters related to the strong seasonality of their sites. These reports were similar with our results suggesting temporal patterns in the SCS might be due to the seasonal currents. Additionally, Gao et al. [38] reported spatial diversity of microbial community in Hawaiian coastal waters and showed coastal waters had the greatest diversity, which was consistent with our study indicating higher diversity in the coastal waters of the SCS. However, none of these studies reported temporal pattern together with spatial pattern, so we do not know which pattern is predominant in sites reported.

Typical marine clades, such as *Alphaproteobacteria*, *Gammaproteobacteria* and *Cyanobacteria* were more represented in marine coastal and open sea samples [36,39], and *Alphaproteobacteria* was more abundant in marine water than in freshwater [40]. In our study, *Alphaproteobacteria* SAR203 was the most abundant clade in marine water samples (Figure 4). The clones were very distantly related to the uncultured organisms that are frequent in mangrove sediments, marine waters, marine sediments [18,41,42]. Interestingly, an OTU D221S78 was only detected in summer samples, which indicated this OTU might be summer-associated species (Figure 4). *Gammaproteobacteria* was one of the dominant phyla in the samples. Being metabolically versatile, this phylum is ecologically very successful [43]. Two clades *Pseudomonas* and *Acinetobacteria* were retrieved from our marine libraries and clustered with sequences retrieved from water of the Sargasso Sea [12], the East China Sea [32], the SCS and North Pacific Ocean [44], marine sediment [18], marine plankton [45] and hot springs [46]. A dominant OTU D1512SP42 mainly existed in spring samples, which suggested this OTU might be spring-associated species (Figure 4, Table 3). These findings indicated that *Alphaproteobacteria* and *Gammaproteobacteria* were widely distributed and formed large cluster in the sea areas surveyed. Our study was different from previous studies that found a high relative abundance of phototrophic *Cyanobacteria* [47,48]. The results revealed *Cyanobacteria* was the most abundant groups in the D221 site and the D224 site (Figure 2), especially in the samples collected at summer (Figure 3c). *Synechococcus* and *Rhizosolenia* were common in the summer waters (Figure 4). The OTU D224SP49 was only detected at the D224 site and could be recognized as a site special species. From the results, it could be suggested that the high abundance and proportion of *Cyanobacteria* were an important feature of the planktonic bacteria in the two sites, and indicated the ongoing deterioration water quality in the area was due to the rapid development of the tourism and sea farming [49].

*Betaproteobacteria* have been commonly detected in freshwater lakes worldwide, where they are the most abundant group [50]. Recovery of 16S rRNA gene clones affiliated to *Betaproteobacteria* is common in libraries constructed from coastal samples [51], but few to no *Betaproteobacteria* have been reported by open ocean surveys [39,52–54]. These findings lead to the idea that bacterioplankton represented by these lineages have a probable freshwater origin and are adapted to coastal marine environments and could be representative bacterioplanckton phylotypes that transit between freshwater and marine habitats [55]. In this study, our data also support this proposal, since *Ralstonia* and *Methylophilus* were detected in our marine libraries, owing to the contribution of freshwater. This result indicates that freshwater bacteria have affected the bacterial community structure of this marine system.

The ecological significance of *Bacteroidetes* has been brought to light because of their proficiency in degrading various biopolymers such as cellulose, chitin and pectin [56]. The *Bacteroidetes* clade was well represented in both saline and freshwater environments [53]. This might be a consequence of the presence of closely related marine phylotypes of common freshwater taxa [48]. In this study, several *Bacteroidetes* related OTUs clustered with sequences from marine habits of different geographic areas, indicating that *Bacteroidetes* are distributed worldwide. A OTU D151S86 clustered with uncultured bacteria was only obtained in summer samples, which suggested this OTU to be summer-associated species.

The number of OTUs identified from D1512SP and D157SP is quite small (Table 2). We thought this was not merely an error in the cloning and sequencing. The D1512SP and D157SP samples were separated from others, and had a positive correlation with the concentration of salinity (Figure 6). However, in spring the China Coastal currents together with the fresh water from the Pearl river and Jianjiang river flowed south-westwards, which contributed to the lower concentration of salinity at the D1512 and D157 site. Due to the lower concentration of salinity, it is not surprising to observe less OTUs in the D1512 and D157 sites.

This study has confirmed that temporal and spatial patterns occur in water bacterial community of the SCS and that the environmental factors governing diversity and structure of bacteria could be identified from the analysis. Due to complicated biogeochemical processes and hydrodynamic conditions, the spatiotemporal diversity of bacterial communities would be very complicated. To better understand the bacterial community structure response to spatiotemporal variations, more intensive microbial measurements using new technologies including metatranscriptomic, metaproteomic approaches should be conducted in future.

## Supporting Information

Figure S1 Rarefaction curves of the bacterial clone libraries (spring, summer, winter). The phylotypes were determined with a 97% similarity cutoff value.
