# Supplementary figures and images for: Temporal and Spatial Diversity of Bacterial Communities in Coastal Waters of the South China Sea
Source: PLoS One. 2013 Jun 13;8(6):e66968. doi: 10.1371/journal.pone.0066968 (PMC3681761; doi:10.1371/journal.pone.0066968)

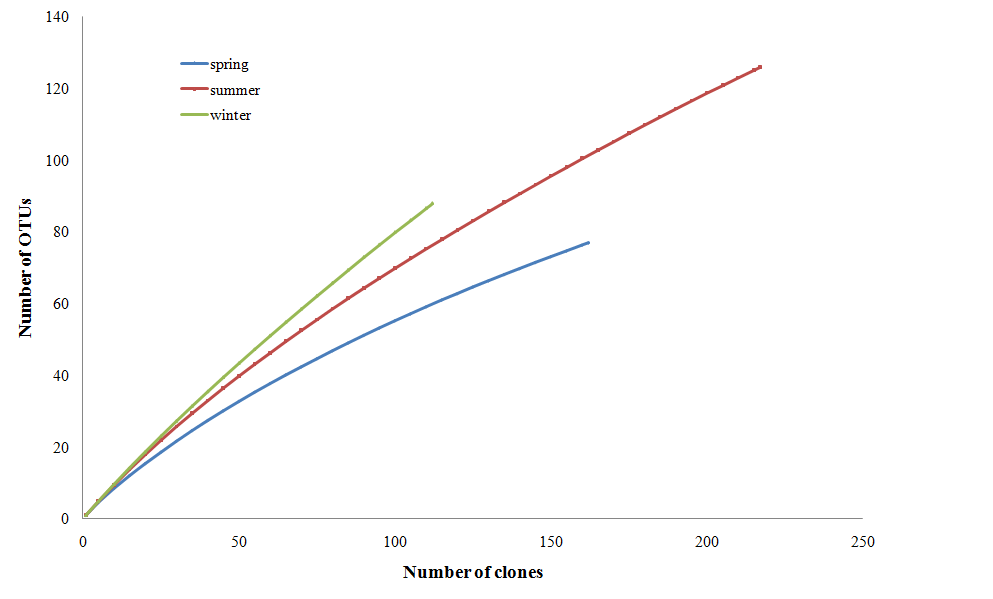

Supplement: Figure S1 — The phylotypes were determined with a 97% similarity cutoff value. [file pone.0066968.s001.tif]
